# De novo KAT6B mutation causes Say–Barber–Biesecker–Young–Simpson variant of Ohdo syndrome in an Iranian boy: a case report

**DOI:** 10.1186/s13256-023-04237-w

**Published:** 2024-01-05

**Authors:** Behzad Davarnia, Mohammad Panahi, Bahareh Rahimi, Hassan Anari, Reza Farajollahi, Ehsan Abbaspour Rodbaneh, Farhad Jeddi

**Affiliations:** 1 Medical Genetics and Pathology, Ardabil University of Medical Sciences, Ardabil, Iran; 2 Department of Medical Biotechnology, Faculty of Advanced Medical Sciences, Tabriz University of Medical Sciences, Tabriz, Iran; 3 Department of Medical Biotechnology, Faculty of Allied Medical Sciences, Iran University of Medical Sciences, Tehran, Iran; 4 Ardabil Welfare and Rehabilitation Organization, Ardabil, Iran

**Keywords:** Say–Barber–Biesecker–Young–Simpson (SBBYS), NGS, KAT6B gene, Human genetics, Case report

## Abstract

**Background:**

Say–Barber–Biesecker–Young–Simpson (SBBYS) (OMIM #603736, Ohdo syndrome variant) is a rare type of severe blepharophimosis intellectual disability syndrome, which is generally characterized by a global developmental delay, distinctive facial features, and intellectual disability with multiple congenital anomalies, including skeletal involvement, missing, or underdeveloped kneecaps, and genital anomalies, in affected males. It has been shown that mutations in the *KAT6B* gene, which is a lysine acetyltransferase-encoding gene, have been associated with SBBYS syndrome. All the known variants are dominant de novo mutations that result in protein truncation.

**Case presentation:**

A 14-year-old Iranian Azeri boy with an intellectual disability, distinct dysmorphic facial features such as open-mouth expression, sparse medial eyebrows, widely spaced upward-slanted eyes, epicanthal folds, broad nasal bridge, low-set ears, anteverted ears, short philtrum, hypertelorism, microphthalmia is presented in this case study. Cryptorchidism was reported. Neurologically, the patient presented with poor eye contact, hypotonia, and speech difficulties. In the skeletal X-ray, underdeveloped kneecaps with some new features were observed.

**Conclusion:**

We present the first case of SBBYS syndrome in association with some new anomaly features in the Iranian population. Based on this diagnosis, we could provide the patient with a suitable plan of management as well as appropriate genetic counseling for his family.

## Introduction

Heterozygous de novo mutations in the *KAT6B* gene, which encodes the histone acetyltransferase, can cause two syndromes with mental retardation and congenital abnormalities, known as Genitopatellar syndrome (GPS; OMIM #606170) and Say–Barber–Biesecker–Young–Simpson syndrome (SBBYSS, OMIM #603736) [1]. The GPS is a severe disease characterized by aplasia, renal anomalies, external genital abnormalities, flexion deformities of the extremities, facial dysmorphism, patellar hypoplasia, microcephaly, agenesis of the corpus callosum (ACC), and severe psychomotor retardation [2–4]. On the other hand, the SBBYSS (a variant of Ohdo syndrome) is a crippling disorder, which is diagnosed based on the presence of distinct facial dysmorphic characteristics, including mask-like face, ptosis, and blepharophimosis. In addition, hypotonia, big toes and long thumbs, dental malformations, feeding problems, hypoplastic or dislocated patellae, hearing impairment, cryptorchidism, congenital heart diseases, severe intellectual impairment, and developmental delay may also be observed in patients with SBBYSS [5, 6]. The majority of the patients reported with SBBYSS were sporadic, but at least three generations affected have been reported in a family [7].

In 1986, SBBYSS was reported by Ohdo *et al*. [8] and then later described in detail as a variant of Ohdo's syndrome by Say and Barber [9], Young and Simpson [10], and Biesecker [11]. SBBYSS is a rare genetic disorder, which has been reported in less than 20 cases in the literature [12]. It is caused by different types of mutations in the *KAT6B* gene [MIM 605880] on chromosomal region 10q22, which encodes a highly conserved lysine acetyltransferase 6B, a member of the MYST (SAS/MOZ) family [13], which plays a remarkable role in chromatin modification, leading to the SBBYSS features and phenotypes.

Because of clinical overlap between GPS and SBBYSS syndromes, the *KAT6B*-related disease spectrum is a subject of interest to researchers [14]. Herein, we are going to report a 14-year-old developmental delay boy with cognitive impairment, significant facial dysmorphisms, undescended testes and bilateral hypoplasis of patellae as a new case of SBBYSS in Iranian population, which is caused by de novo mutation in *KAT6B* gene.

## Case presentation

### Evaluation of clinical features in the proband

The proband is an Azeri boy of 14 years old from a healthy and non-consanguineous couple. Intrauterine growth retardation was identified in the pregnancy of the proband. He was born at 36 weeks by cesarean section due to no fetal movement, with Apgar scores 9/10. At birth, he was 2.97 kg (50th percentile, − 0.45 SD) and his cranial circumference was 33 cm (10th percentile, − 0.61 SD). The subject began to sit at four years of age and made his first unaided steps when he was six. He could take some steps alone and walk with an increased lift base. At nine years of age, he was referred to a medical genetic counseling center and diagnosed with blepharophimosis syndrome because of blepharophimosis and intellectual impairment. He showed a few episodes of loss of consciousness at six years old, but his electroencephalogram (EEG) was normal. He presented a non-symptomatic large atrial septal defect, ASD (ostium secundum atrial septal defects).

**Fig. 1:**
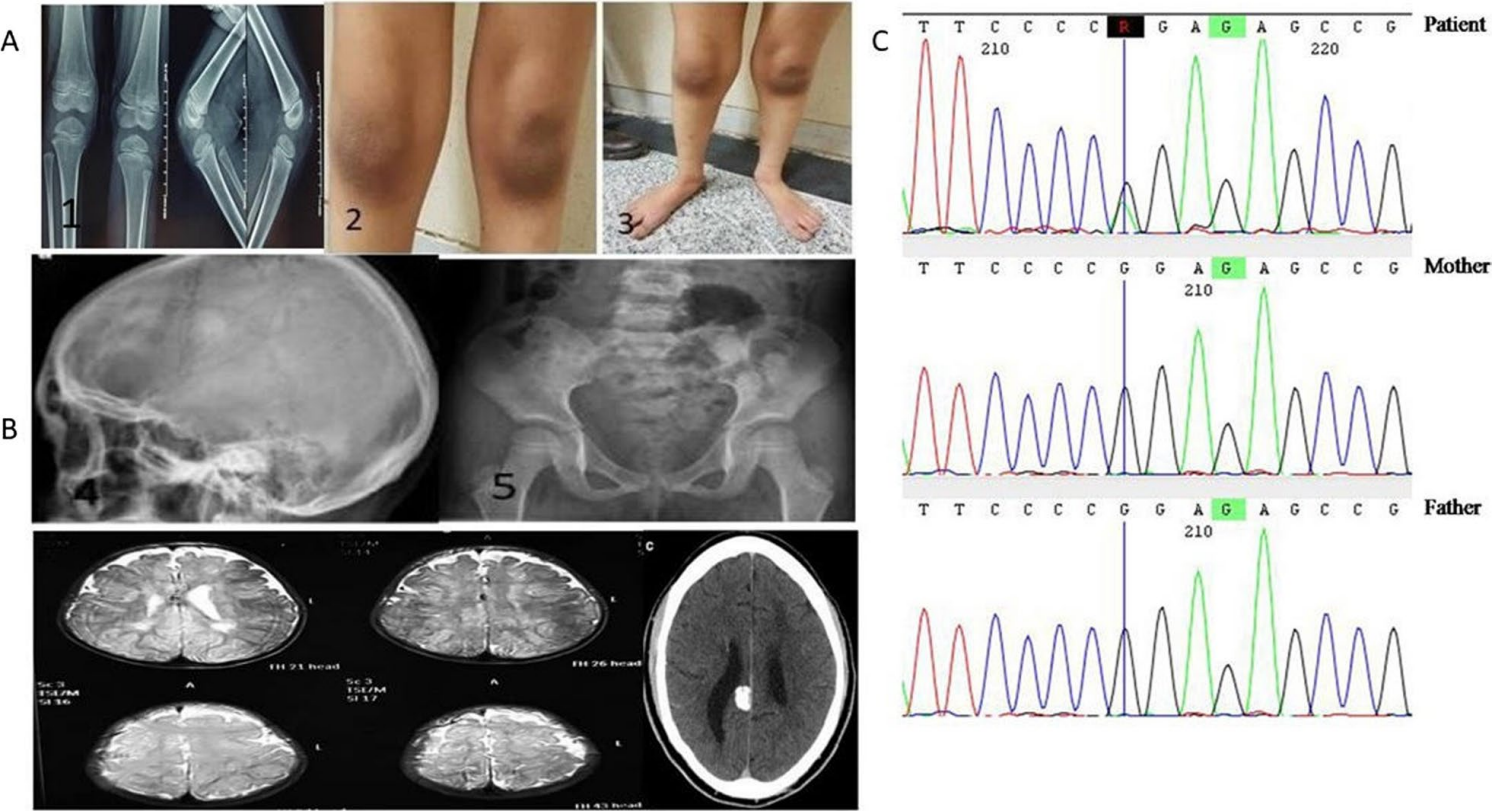
**A** Phenotypic characteristics of the patient; **B** Brain MRI of the patient. Imaging of the brain shows inappropriate myelination and disturbed white matter integrity; **C** Genetic test results. Sanger sequencing reveals a c.3147G>A; p.P1049P de novo heterogeneous mutation in this patient

On physical examination at 9 years old, the patient occipitofrontal circumference (OFC) was 52cm (~ 50th centile) but had dysmorphic facial features including flat occiput, triangular long face, frontal bossing, protruding tongue that caused an open mouth expression, a thin upper lip, sparse medial eyebrows, thin eyebrow, epicanthal folds, widely spaced upward-slanted eyes, broad nasal bridge, low-set ears, anteverted ears, short philtrum, hypertelorism, ptosis, microphthalmia, hypoplastic teeth. Congenital urogenital anomaly as cryptorchidism without any renal anomalies was diagnosed. The patient was tall stature and had first (big) toe/long thumbs with patella missing but the most significant abnormal findings in skeletal and joint X-ray, which have not been reported so far, included symmetric bilateral coxa valga, hypoplastic iliac wings and scoliosis, increased diameter of the lumbar vertebral canal, genu valgum, pes planus and sloping forehead with a large brain capacity (Fig. 1A).

From the neurological point of view, he presented with global developmental delay, intellectual disability, poor eye contact, hypotonia and speech difficulties. Brain MRI (magnetic resonance imaging) showed no evidence of corpus callosum agenesis, however inappropriate myelination and disturbed white matter integrity was the significant findings (Fig. 1B). There was no sphincter control. Now, the patient is taken care of mental disability care institute on physiotherapy and speech therapy, due to inability of parents to take care and behavioral disturbances and ADHD (attention deficit-hyperactivity disorder) traits, respectively. In Table 1, we compared the clinical presentations of the present patient with that of some patients in previous reports.

**Table 1:** Comparison of our clinical presentation with that of other patients and their respective reports

	Present study	Yilmaz *et al*. (patient 1) [1]	Yilmaz . (patient 2) [1]	Yilmaz . (patient 3) [1]	Marangi *et al*. [24]	Mendez *et al*. [20]	Sun *et al*. [25]	Shin *et al*. [19]
Mutation	c.3147G>A; p.P1049P	c.3147G>A; p.P1049P	c.3147G>A; p.P1049P	c.3147G>A; p.P1049P	c.1045_1049delTTAAA, p.L349Afs*9	c.4572_4573dup (p.(Thr1525Ilefs*25))	c.3185del (p.leu1062Argfs*52)	c.5206C>T (p.Gln1736Ter)
Publication year	−	2015	2015	2015	2017	2020	2023	2021
Country	Iran	Hungary	Portuguese	UK	Italy	Argentina	China	Korea
Sex	Male	Female	Female	Female	Female	Male	Male	Male
Speech difficulties/delay	+	+	+	+	+	NA	+	+
Growth retardation	+	+	+	+	NA	NA	+	+
Blepharophimosis	+	+	+	+	NA	+	NA	+
Intellectual impairment	+	+	+	+	+	NA	+	+
Visual impairment	+	+	+	−	+	+	−	NA
Heart Defects	+	−	+	NA	−	+	+	+
Genital anomalies	−	−	−	−	−	+	−	NA
ASD	+	NA	NA	NA	NA		NA	+
Dysmorphic facial feature	+	+	+	+	+	+	+	+
Skeletal anomalies	+	NA	NA	NA	NA	NA	NA	NA
Thyroid anomalies	−	+	+	NA	+	NA	NA	+
Hypotonia	+	+	+	NA	+	+	NA	+
Reference	−	(1)	(1)	(1)	(2)	(3)	(4)	(5)

*ASD* ostium secundum atrial septal defects

### DNA extraction and quality control

In the present study, written informed consent was obtained from the parents of a 14-year-old male with SBBYS syndrome; then, he was included in the study. The blood sample was collected, and DNA was extracted by standard salting-out method. Finally, the integrity and purity of DNA were analyzed by agarose gel electrophoresis and Nanodrop spectrophotometer (Thermo Fisher Scientific, Waltham, MA, USA), respectively [15].

### Whole exome sequencing and data analysis

After extraction of Genomic DNA from the proband, it was processed for whole-exome sequencing (WES) using the SureSelect Human All ExonV6 kit. The DNA was fragmented, and blunt ends were created. Adapter oligonucleotides were ligated to DNA fragments, followed by enrichment via PCR. Purification was performed using the AMPure XP system, and quantification was done using the Agilent Bioanalyzer 2100. The captured library was sequenced on a NovaSeq 6000 Illumina sequencer with an average coverage of 100X. Data quality was assessed using FastQC and NGS QC toolkits. High-quality reads were aligned to the human reference genome (GRCh37.p13/hg19) using the Burrows-Wheeler Alignment tool. Picard tools were employed to mark and remove duplicated reads, and the SAM file was converted to BAM format. Base quality score recalibration was performed using GATK. Variants were filtered based on criteria such as minor allele coverage ≥ 10, variants with coverage ≥ 15, and call quality ≥ 20. ANNOVAR was used for genetic variant annotation. Annotated variants were further filtered and selected for analysis.

This process enabled the identification and analysis of genetic variants in the proband's genomic DNA sample [16].

### Functional analysis of the detected mutation

The pathogenicity of the identified variant was assessed through in silico analysis using web server Mutation Taster. Prediction of the structure of wild-type and mutant protein was done by SWISS-MODEL, the SWISS-MODEL web server (https://swissmodel.expasy.org/). To predict whether the identified variant would escape NMD, the nonsense-mediated mRNA decay (NMD) ESC predictor was used (https://nmdprediction.shinyapps.io/nmdescpredictor/). Also, the STRING database was used to predict interactions among proteins.

### Sanger sequencing for validation of variants

After WES, Sanger sequencing was used to validate the variant through specific primers. According to the reference genomic sequences from the Human Genome from GenBank in NCBI, primer pairs were designed for the candidate loci (Fig. 1C). We identified a de novo heterozygous mutation of guanine to adenine *NM_012330.4(KAT6B):c.3147G*>*A (p.Pro1049* =*)* using WES*.* Sanger sequencing subsequently confirmed this heterozygous variant identified by WES in this individual. The mutation could not be detected in the parental DNA by Sanger sequencing (Fig. 1C), suggesting its de novo occurrence. The results of sequence analysis revealed that the c.3147G>A; p.P1049P transition at codon 1,049 was a synonymous mutation (p.Pro1049 =) in the exon 16 of the *KAT6B* gene.

**Fig. 2:**
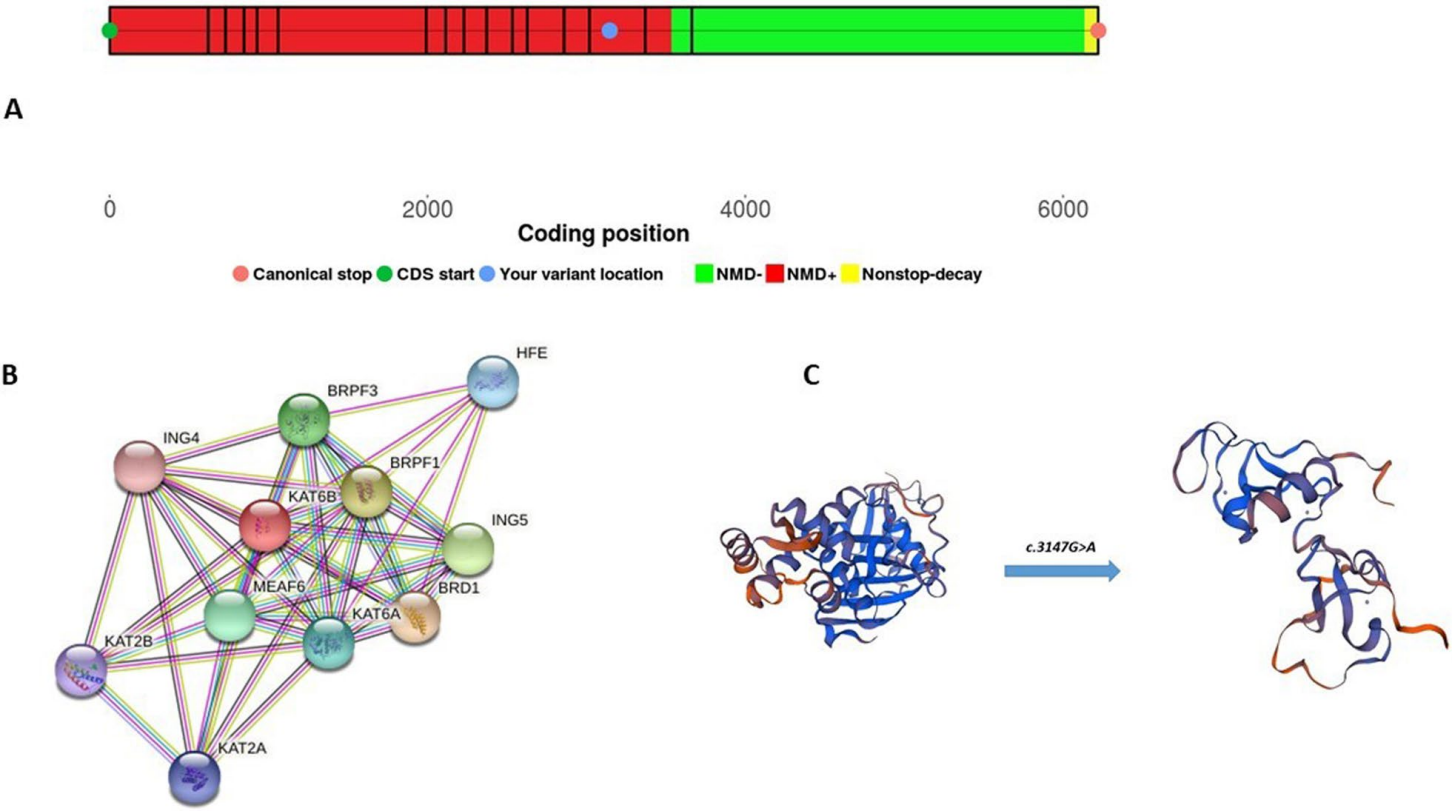
**A** The result of nonsense-mediated mRNA decay prediction in the patient by the NMD Esc Predictor Web Site. **B** Protein–protein interaction network of *KAT6B* gene. **C** Prediction of the effect of p.P1049P mutation on the protein structure and chains using Swiss model

In addition, KAT6B protein is a 231,378 Da protein with 2,073 aa. Functional annotation of *KAT6B* by DAVID displayed that this protein expresses in different human tissues. Considerably, it has high quantities in the immune cells, uterus, thymus, fetal brain, and other tissues according to the (http://biogps.org). Also, KAT6B protein has been identified in different diseases such as genitopatellar syndrome, ohdo syndrome, Sbbyss variant, monocytic leukemia, and blepharophimosis. Furthermore, the NMD web server represented this premature codon likely led to nonsense-mediated degradation (Fig. 2A). According to the InterProScan as a tool for the analysis of protein domain, *KAT6B* encodes a histone acetyltransferase protein with the N-terminus which is involved in transcriptional activation and the C-terminus, which play a role in transcriptional repression.

The analysis of interaction among proteins by STRING indicated that KAT6B protein creates a network with about some different gene products (Fig. 2B). Also, this protein plays an important role in some biological pathways through three important activities, including histone acetylation, nucleosome assembly, and regulation of transcription (https://www.ebi.ac.uk/QuickGO/GTerm). The SWISS-MODEL server displayed this mutation resulted in a truncated protein (Fig. 2C).

## Discussion

Say–Barber–Biesecker–Young–Simpson (SBBYS) is a rare genetic abnormality due to the mutations in the *KAT6B* gene. The *KAT6B* encodes a histone-acetyl transferase, which is a highly conserved part of the MOZ/MORF complex. In addition to the activity of acetyltransferase, C-terminal and N-terminal ends of the encoded protein have transcriptional repression and transcriptional activation activity, respectively. This protein is essential for RUNX2-dependent transcriptional regulation and can be involved in the growth of the brain. Because of the clinical overlapping and similarities, some researchers proposed that *KAT6B* causes two allelic GPS and SBBYS syndromes, whereas others have suggested them as two different disorders [14, 17]. Although SBBYSS and GPS have been described as two distinct genetic disorders, they have some overlapping phenotypes, including genital abnormalities, hearing loss, global developmental delay, hypotonia, congenital heart defects, intellectual disability, and thyroid anomalies [17]. Lemire *et al*. [18] suggested some features might raise for *KAT6B*-related anomalies. Patients with two major phenotypes or one major phenotype and two minor phenotypes presumably have a *KAT6B-*related disorder. The main phenotypes of SBBYSS are described by mask-like facial appearance, long thumbs/great toes, blepharophimosis, dacryostenosis, and ptosis. However, the distinct features of GPS are characterized by agenesis of the corpus callosum, club feet, patellar hypoplasia/agenesis, and flexion contractures of the hips and knees [19]. Nevertheless, it is noticeable that Mendez *et al*. [20] reported a patient with long thumbs/halluces and dysmorphic face as the major features, suggesting the identification of SBBYSS, while he had a corpus callosum agenesis, which is a major phenotype of GPS. Also, Niida Yo *et al*. [21] reported a case with *KAT6B* mutation that was formerly reported in SBBYSS cases, but he also had some features, including genital anomalies, laryngomalacia, and developmental delay that confirmed GPS. Our patient has some distinct major features, including dysmorphic facial and blepharophimosis with big toes and long thumbs, suggesting the SBBYSS. In addition, we identified some new skeletal abnormality features and phenotypes, including symmetric bilateral coxa valga, hypoplastic iliac wings and scoliosis, and increased diameter of the lumbar vertebral canal, genu valgum, and pes planus that had not been reported in previous cases to date altogether. It is noticeable that *KAT6B-*related disease spectrum, especially GPS and SBBYSS have clinical similarities and overlapping in their phenotypes, and making a clear genotype–phenotype correlation is very challenging, if not possible. Although an appropriate genotype–phenotype correlation is required for accurate diagnosis, a clear correlation has not yet been defined. We hope that these new phenotypes and features help make appropriate and credible genotype–phenotype correlations to identify *KAT6B*-related disorders.

Furthermore, our results are comparable with two different studies. The mutation has been previously reported as a pathogenic variant by Gannon *et al*. and Yilmaz *et al*. in six patients with SBBYSS [1, 22]. By using Alamut 2.2 prediction program, Gannon *et al*. proposed that c.3147G>A; p.P1049P mutation could lead to a cryptic splice acceptor site, which could be followed by protein truncation. They suggested that this mutation could be disease-causing; however, no experimental evidence was available in support of aberrant splicing. Clinically, they indicated that all three patients with that splice site variant were fitted better with SBBYSS [22]. In another study, to experimentally evaluate this aberrant splice site change, Yilmaz *et al*. extracted total RNA from blood sample of patient and their healthy parents. Then, reverse transcriptase PCR (RT-PCR) was applied with specific primers for amplifying the part of exons 15 and 16 of the *KAT6B* gene, followed by sequencing analyses of PCR products. They showed that aberrant splicing of *KAT6B* could result in an out-of-frame deletion of 127 bp in exon 16, which was followed by a premature stop codon (p.Ala1008Argfs 62) [1].

Our genetic findings confirm the diagnosis of SBBYSS in the 14-year-old boy due to a known de novo heterozygous mutation in *KAT6B* gene. Since already seven unrelated individuals reported c.3147G>A; p. p.P1049P is actually the most common *KAT6B* mutation, which is detected in individuals with SBBYSS. In addition, our results are in line with previous studies that suggested that the position of the mutations in *KAT6B* is associated with the phenotypes of the patients. Previous studies indicated that most mutations in *KAT6B* were in exon 18, which is the last exon of *KAT6B* and encodes the highly conserved C-terminal acidic and transcriptional activation domains [7, 17]. Gannon *et al*. [22] suggested that while GPS mutations occurred more proximally in the exon 18, losing both acidic and transcriptional activation domains led to gain-of-function mutation and affected the critical binding sites of the protein. SBBYSS mutations happened more distally in the same exon, lacking the transcriptional activation domain and preserving the acidic domain that led to loss-of-function mutation. Also, it is noticeable that recent studies reported mutations more proximal in exons 15, 16, and 17 with the typical phenotypes of SBBYSS [1]. As a result, screening of exon 16 along with exons 15, 17, and 18 have to be tested in targeted mutation detection methods for diagnosis of the *KAT6B-*related spectrum abnormalities due to its recurrence [23].

## Conclusion

In the current case study, a de novo heterozygous mutation was detected in the *KAT6B* gene in an Iranian male manifesting typical SBBYS symptoms. The c.3147G > A; p.P1049P transition at codon 1,049 was a synonymous mutation, which resulted in a cryptic splice acceptor site. Our patient is the first case report due to *KAT6B* alteration in Iranian patients. Records of further evidence and patients with GPS and SBBYSS phenotypes can support accurate identification and describe genotype–phenotype correlation.

## Data Availability

Input data for the analyses are available from the corresponding authors on request.
